# Flavored Tobacco Sales Restrictions Reduce Tobacco Product Availability and Retailer Advertising

**DOI:** 10.3390/ijerph19063455

**Published:** 2022-03-15

**Authors:** Louisa M. Holmes, Lauren Kass Lempert, Pamela M. Ling

**Affiliations:** 1 Departments of Geography and Demography, The Pennsylvania State University, University Park, PA 16802, USA; 2 Center for Tobacco Control Research and Education, University of California San Francisco, San Francisco, CA 94117, USA; lauren.lempert@ucsf.edu (L.K.L.); pamela.ling@ucsf.edu (P.M.L.)

**Keywords:** prevention, tobacco control, health policy, youth

## Abstract

Objective: This study examined differences in the availability and advertising of flavored tobacco products before and after flavored tobacco sales restrictions were enacted in Alameda and San Francisco Counties in California. Main outcome measures: Data were collected from a sample of tobacco retailers in Alameda and San Francisco Counties at two time points: 2015, before flavored tobacco policies were enacted, and in 2019–2020, after some cities had enacted policies. Retailers were separated by city into Category 1 (*n* = 442)—retailers in cities that enacted a flavored tobacco policy between the two data collection periods, and Category 2 (*n* = 89), those that had not. Means comparison tests were conducted to evaluate significant differences over time and by category. Results: There was significantly reduced availability of menthol cigarettes, flavored little cigars, smokeless tobacco, vape pens, and Blu brand menthol e-cigarettes between 2015 and 2020 in Category 1 retailers. Category 2 retailers had reduced availability only for Blu menthol e-cigarettes and demonstrated an increase in smokeless tobacco availability. Exterior store advertising for cigarettes, little cigars, cigars, and e-cigarettes also decreased significantly in Category 1 cities relative to Category 2 cities; 8.1% of Category 1 stores were advertising flavored tobacco products in 2019–2020 compared to 36.2% of Category 2 stores. There was also a 78% reduction in flavored ads between 2015–2019 in Category 1 cities compared to a 38% decrease in Category 2 cities. Tobacco advertising inside Category 2 stores increased. Finally, Category 2 cities had significantly greater availability of cigalikes, mod or tank vapes, flavored e-cigarettes, and e-liquids compared to Category 1 cities. Conclusions. Comprehensive flavored sales restriction policies reduce flavored tobacco availability and tobacco advertising, which may help prevent youth tobacco initiation and exposure.

## 1. Introduction

Flavored tobacco products appeal to youth and are a leading driver of youth initiation of tobacco products [1]. Since menthol-flavored products create a cooling sensation and are perceived as less harmful, they pose an additional public health threat by increasing the likelihood that youth and young adults will begin smoking [2]. Menthol products have also had a disproportionate health impact on African-Americans due to the tobacco industry’s targeted marketing [3]. In 2009, federal law prohibited “characterizing flavors” in cigarettes, but exempted menthol. Nor did the law prohibit flavors in other tobacco products, such as cigars, little cigars, smokeless tobacco, hookah, and electronic cigarettes (e-cigarettes) and e-liquids [4]. While this law led to declines in adolescent cigarette use, it was associated with increases in teen use of menthol cigarettes and other tobacco products featuring sweet, fruity, and mint flavors [5].

The national tobacco landscape changed dramatically between 2015–2020. While e-cigarettes were first introduced to the market in 2006, major sales by any e-cigarette brand were enjoined until 2011 when, after a series of court battles, FDA announced it would regulate e-cigarettes in the same manner as tobacco products from that point forward [6]. That opened the door for brands like NJOY and Blu to begin widespread sales and marketing of cigalikes, vape pens, and rechargeable e-cigarettes [7]. By 2015, e-cigarette technology had evolved, and the pod vape, JUUL, was introduced to the market in several flavors. JUUL was aggressively advertised to and became popular with young people [8]. By December 2018, JUUL accounted for about 75% of the US e-cigarette market. Other pod-based flavored e-cigarettes (“JUUL-alikes”) also flooded the market [9].

Following a national outcry over this “epidemic” of youth e-cigarette use, in 2018 JUUL removed most of its flavored products (excluding tobacco and mint) from brick-and-mortar stores [10], and in 2019 JUUL stopped selling mint-flavored e-cigarettes. In January 2020, the US Food and Drug Administration (FDA) announced it would prioritize enforcement against unauthorized flavored cartridge-based e-cigarettes, but exempted flavored disposable and menthol- and tobacco-flavored e-cigarettes [11]. Following FDA’s action, sales of menthol-flavored cartridge-based and flavored disposable e-cigarettes increased [9,12]. FDA announced in April 2021 that it planned to initiate rulemaking to prohibit sales of menthol cigarettes and flavored cigars [13], but implementation of federal proscriptions could take years to complete. To address gaps in federal law, states and local jurisdictions have passed laws regulating flavored tobacco product sales. As of September 2020, eight states, 325 localities, and five Indigenous nations had passed laws restricting flavored tobacco product sales [14].

Studies of local laws restricting flavored tobacco product sales in Canada [15], New York [16], Minneapolis and Saint Paul [17], Providence [18], Chicago [19], and Massachusetts [20,21] have found varying success in reducing product sales or availability. The comprehensiveness and types of flavors included in these policies varied, leading several researchers to conclude that more comprehensive flavor bans, especially those including menthol, have greater potential to reduce tobacco use and health disparities [5,15,22]. Comprehensive local laws, i.e., those that prohibit all flavored tobacco product sales, including menthol, have the potential to reduce the availability of flavored tobacco products in retail environments, ultimately helping to reduce youth tobacco initiation. They further have the potential to reduce tobacco advertising as stores that no longer sell flavored products are not likely to advertise those products either. One of the first comprehensive policies eliminating sales of all flavored tobacco products, including menthol, was passed in San Francisco in 2018 and enforced in 2019 after a year during which the San Francisco County Department of Public Health trained and educated tobacco retailers on the new policy’s requirements [22].

**Figure 1:**
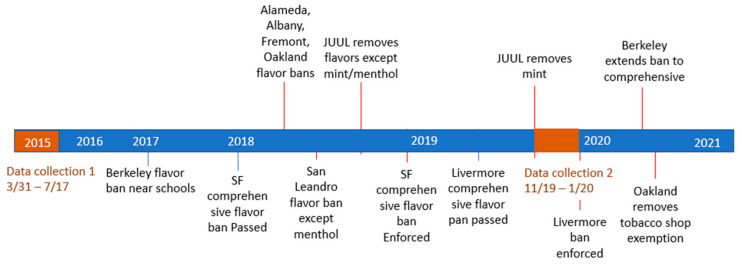
Brief Timeline of Tobacco Retail Data Collection Periods and Relevant Flavored Tobacco Policy Changes.

In addition, cities in Alameda County, CA implemented a varied set of flavored tobacco sales restriction policies between 2014 and 2020 (Figure 1), creating a natural experiment within the San Francisco Bay Area (Bay Area). To the best of our knowledge no papers have compared the availability of flavored tobacco products in Bay Area cities with comprehensive flavor policies (prohibiting retail sales of all flavored tobacco products, including menthol) to availability in cities with partial flavor bans (exempting menthol or limiting sales only in distance of schools) or to cities that have not adopted flavored tobacco policies of any kind. Additionally, we found no papers that investigated the impact of Bay Area flavored tobacco policies on tobacco retailer advertising.

**Figure 2:**
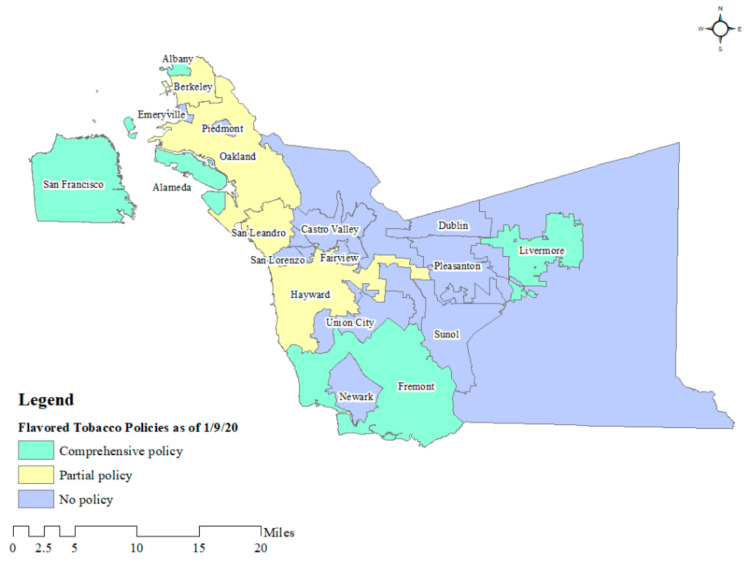
Alameda and San Francisco County Cities Color-Coded According to Comprehensiveness of Flavored Tobacco Sales as of January 2020.

In the San Francisco Bay Area, 14 jurisdictions have passed ordinances restricting sales of flavored tobacco products, 13 of which are comprehensive policies that include menthol, as shown in Figure 2 [14]. This study analyzes data collected in 2015 (before implementation of any flavored tobacco restrictions, except a limited policy in Hayward) and in 2019–2020 (after implementation of numerous flavored tobacco policies) from tobacco retailers in the City and County of San Francisco and cities across Alameda County to determine the extent to which local flavored tobacco sales restrictions affected the availability of flavored tobacco products. We compared flavored tobacco product availability in cities with comprehensive flavored sales restrictions that were enacted between 2015 and January 2020 when we collected our two waves of retail data (Figure 2) [23], to cities that did not have a comprehensive policy in place as of our second wave of data collection. As of July 2021, three cities in Alameda County had not yet passed any flavored tobacco policy (Emeryville, Newark, and Union City). We hypothesized that flavored product availability would be less in cities with comprehensive policies compared to those without. Figure 2 illustrates policy coverage by city as of 9 January 2020, when we completed our second wave of tobacco retail data collection. We additionally analyzed the impact of local flavored sales restrictions on tobacco retailer advertising, hypothesizing that a reduction in sales of various flavored products would be associated with a reduction in advertising for those products.

## 2. Materials and Methods

### 2.1. Tobacco Retailer Samples

Data from tobacco retailers in Alameda and San Francisco Counties were collected as part of a larger study involving a probabilistic household panel survey of young adults in the study region [24]. The first wave of tobacco retail data collection took place in 2015, followed by the second wave in 2019–2020. Both tobacco retail samples drew from lists of all licensed tobacco retailers in the two counties supplied to us by the Alameda and San Francisco County Departments of Health. The sampling method differed slightly between 2015 and 2019 due to changes in the nature of the overall research project. One goal of the retailer audits was to evaluate tobacco use behavior among young adults in relation to retail exposure; therefore, our retail sample frames were partially devised based on the geographic distribution of our young adult survey participants. However, participants were widely distributed across the two counties, and the store types were similar in each time point. Specifically, 37% of stores in 2015 were convenience stores compared to 35% in 2019–2020, 30% were small groceries/delis in 2015 compared to 23% in 2019–2020, 5% in each time period were supermarkets, and tobacco shops represented 6.2% versus 9% of retailers in 2015 compared to 2019–2020. The only category with a notable difference was liquor stores, representing 14% of stores in 2015 compared to 26% of stores in 2019–2020. It is unclear whether this change was due to sampling, or a change in the composition of retailers between the two time periods. There were 38% fewer tobacco retailers in 2019–2020 compared to 2015, so it may be that liquor stores were more likely to keep selling tobacco.

For the 2015 data, we sampled 100% of retailers located in the Census block groups where participants in our corresponding household survey resided and added a five percent simple random sample of remaining retailers (*n* = 253 of 2353, or 11% of stores). For the 2019–2020 tobacco retail audit, data were collected simultaneous to data collection for our corresponding household survey. Our tobacco retailer sample was therefore drawn based on: (1) residential location of baseline survey respondents; (2) retailers from the 2015 sample that were still in business and could be revisited in 2019–2020; and (3) a supplemental proportional random sample by city of remaining tobacco retailers. We ultimately visited 369 tobacco retail stores in this wave (of 1707 total or 22%), 29 of which had also been visited during Wave 1. In Wave 2, 161 of the 369 retailers sold e-cigarettes; the analysis of Electronic Nicotine Delivery Systems (ENDS) availability is limited to these retailers.

### 2.2. Measures

In both waves of data collection, a questionnaire adapted from the Standardized Tobacco Assessment for Retail Settings was used [25]. This was programmed in Qualtrics for 2015 data collection, which took place between 31 March and 17 July of that year and using ESRI’s Survey123 software for the latter data collection, which occurred between 4 November and 9 January of 2019–2020. Research Assistants (RAs) attended a full day training to orient them to the context of the study and data collection methods, survey measures, and how to recognize diverse tobacco products, brands and flavored variants, and advertising/promotion. They further learned how to collect pricing data, including practicing field data collection using the study instruments and protocols. RAs were equipped with smart phones and tablets for field data collection. They visited each tobacco retailer in pairs to obtain information on tobacco product advertising, availability, and pricing. This paper focuses on tobacco advertising enumerated for both store exteriors and interiors and the availability of various flavored tobacco products.

Exterior tobacco advertising was measured by asking whether or not non-menthol cigarettes, menthol cigarettes, little cigars, cigars, smokeless tobacco (chew, snus, dip, or snuff), e-cigarettes, JUUL, or other pod vapes were “advertised OUTSIDE the store (on windows/doors, building, sidewalk or elsewhere).”

Interior tobacco advertising was measured with two questions: (1) Please indicate the level of advertising of tobacco products (excluding e-cigarettes) in the store; and (2) Please indicate the level of advertising of e-cigarettes in the store. Responses were on a scale from 1 (No ads) to 4 (Ads everywhere you look).

Product availability was ascertained with “yes” or “no” responses once a store was confirmed to sell tobacco products, including whether flavored versions of little cigars, smokeless tobacco, and e-cigarette products were sold. Two specific products were identified—Newport menthol cigarettes and Blu menthol e-cigarettes—to ensure price comparability across stores, but other questions were asked about broadly, e.g., “does this store sell little cigars?”

Policy category is a binary variable indicating cities that (1) had enacted flavored tobacco sales policies between data collection waves one (2015) and two (2019–2020), and (2) those that enacted such policies after our second wave of data collection or did not have any policy in place. Most of the policies in Category 1 were comprehensive, with three exceptions: Berkeley initially passed legislation restricting flavored tobacco sales within 600 feet of schools, San Leandro exempted menthol, and Oakland exempted “tobacco stores,” in which tobacco sales constituted 60% or more of the store’s revenue. Berkeley made its policy comprehensive in April 2020, and Oakland closed its exemption in May 2020, but both policy changes occurred after our second wave of data collection [26]. Hayward passed a flavored sales restriction policy in 2014, but it only applied to tobacco retailers who obtained a license after the date of policy enactment. Of the 33 stores sampled in Hayward, only four were not selling flavored products. Therefore, the latter four stores were included in category 1 while the remaining stores were included in category 2.

### 2.3. Analysis

Using Stata v16, we conducted means comparison tests between the two data collection periods, 2015 and 2019–2020, for those cities with and without flavored tobacco policies as of 9 January 2020, to ascertain whether there were significant differences in product availability and advertising over time based on policy enactment. We also evaluated differences in ENDS and associated products between the cities with and without flavored tobacco policies for the 2019–2020 timeframe. As many of these products were not on the market during our first wave of data collection, we only collected this information in the second wave.

## 3. Results

Table 1 shows frequencies and confidence intervals for flavored tobacco product availability in our initial wave of data collection in 2015 and our second wave in 2019–2020, first in cities that had flavored tobacco policies in place (policy Category 1) and then in those that did not (policy Category 2) as of 9 January 2020. In general, most products became less available in policy Category 1 and remained the same or became more available in policy Category 2. Specifically, there was a significant reduction in the availability of Newport menthol cigarettes, from 89.9% in 2015 to 19.6% in 2019–2020 in the cities with policies, whereas there was no statistical change in the other cities. There was also significantly reduced availability of Newport cigarettes in Category 1 compared to Category 2. In Category 1, there were also significant reductions in flavored little cigar (90.5% of stores that sold little cigars to 22.4%), smokeless tobacco (83.5% of stores that sold smokeless tobacco to 25.0%), vape pen (19.2% to 9.4%), and Blu menthol e-cigarette sales (53.3% to 6.2%) over time. There was also significantly less availability of flavored little cigars, smokeless tobacco in general, and flavored smokeless products, as well as e-cigarettes and Blu menthol e-cigarettes. Non-flavored little cigars saw no significant change over time or between categories, and while vape pens were less available over time in Category 1 cities, there was no difference when compared with Category 2 cities. There were two significant changes in Category 2 cities over time, one of which was an increase in availability of smokeless tobacco products from 55.9% to 79.6% of stores. Additionally, Blu menthol e-cigarette sales dropped from 64.3% to 34.3%.

Table 2 shows tobacco product advertising intensity on store exteriors and interiors at wave one and wave two, separated by policy category. Category 1 cities had significant reductions in exterior advertising for non-menthol cigarettes (47.6% to 25.4%), menthol cigarettes (39.3% to 6.4%), little cigars (25.7% to 12.3%), cigars (6.8% to 3.0%) and e-cigarettes (32.0% to 9.7%) between time points, as well as interior tobacco (60.9% to 40.4%) and e-cigarette (39.6% to 19.3%) advertising. In Category 2 cities, advertising frequency dropped significantly for e-cigarette advertising (41.7% to 20.4%), whereas interior tobacco advertising frequency increased (50.0% to 79.2%) over time. Exterior advertising for cigarettes, both menthol and non-menthol, smokeless tobacco, and pod vapes other than JUUL was statistically higher in Category 2 compared to Category 1 cities, as was interior tobacco advertising.

Table 3 shows differences in ENDS and associated product availability by policy category only as pod vapes were not widely available in 2015. Cigalikes were more likely to be sold in Category 2 cities (64.9% vs. 46.8%) as were mod or tank vapes (21.1% vs. 10.6%), flavored e-cigarettes (86.8% vs. 21.3%), and e-liquids (23.7% vs. 12.7%). JUUL products, whose cartridges come in “Virginia Tobacco” flavor, remained available in many stores, regardless of category.

## 4. Discussion

Retail availability of flavored tobacco decreased significantly between July 2015 and January 2020, driven by localities with comprehensive sales restriction policies (Figure 2; Supplementary Table S1). Overall, the availability of flavored tobacco decreased by 90.6% in cities with comprehensive flavored tobacco sales restrictions and 62.7% in cities with partially restrictive policies, compared to 13.6% in cities without any policy at the time we completed data collection. This latter reduction in flavored product availability may be due to general movements in the tobacco market, anticipation of pending flavored tobacco legislation, or spillover effects from flavor policies in neighboring cities. For example, the city of Newark, which had no policy, is surrounded by Fremont (Figure 2), which had a comprehensive policy.

These data suggest that flavored tobacco policies were implemented effectively in both counties, but to differing degrees. San Francisco County saw a drastic reduction in flavored product availability and associated tobacco and ENDS advertising between 2015–2020 as well as in comparison to cities without flavored sales policies, while Alameda County saw less dramatic reductions, likely due to inconsistent policy enactment across cities. These findings are consistent with prior studies that found high levels of policy adherence in San Francisco following enforcement of the ordinance [27,28], and incomplete adherence in Oakland based on littered cigarette packs collected from 15 census tracts in Oakland, which found 46% of cigarette packs were menthol [3]. However, the current study measured availability through direct retail audits rather than pack litter, which might include menthol cigarette packs acquired from outside the study area. The current study also extends prior work, which was limited to cigarettes or cigars, or which compared San Francisco to other cities with no policy [28], to find significant reductions in the availability of menthol cigarettes, flavored little cigars, smokeless tobacco and flavored ENDS products in Category 1 cities compared to those with partial or no policies.

Together these findings suggest that exceptions in flavored tobacco policies may decrease effectiveness and that partial policies may not address disparities generated by decades of targeted menthol marketing aimed at African-Americans [3,18,29,30]. The population composition of the cities with partial policies underlines this trend; Black people represented 13.6% of Berkeley, Oakland and San Leandro’s population compared to 4.0% of the population in the cities with comprehensive policies [29]. Latinx populations too have been a target of menthol advertising [30], and Latinx youth have demonstrated increasing rates of e-cigarette use in recent years [31,32,33]; the percentage of Latinx individuals covered by a comprehensive flavored tobacco policy was 15.1% compared to 21.8% in cities with partial restrictions, and 23.5% in cities without any policy [29].

The differences between Category 1 and Category 2 cities and prevalence rates over time might be due to several factors: in San Francisco, for example, a year of retailer outreach, engagement, and education took place prior to enforcement of the policy [27], and since the city and county are congruent administrative units, the policy implementation efforts applied consistently. In contrast, different cities in Alameda County had different policies, exemptions, times of implementation, and fewer resources for retailer outreach, so that Category 2 cities may have had more variable implementation and enforcement of the policies passed. Additionally, three cities in Category 2 did not have any flavored tobacco policy as of this writing. However, cities with partial policy restrictions still showed significant decreases compared to those cities that lacked any policy. Taken together, these data suggest that the implementation of comprehensive policies eliminating sales of flavored tobacco was associated with a significant reduction in flavored product availability, with the strongest effect in the locations with the most comprehensive implementation. Thus, strong statewide or federal policies eliminating sales of flavored tobacco products implemented in a consistent manner with retailer education, engagement, and outreach are likely to reduce flavored tobacco availability.

The second important finding of this study was a significant decrease in tobacco advertising between the two study time points, and between the cities with comprehensive policies versus those without, for almost all tobacco products, including menthol and non-menthol cigarettes, cigars, little cigars, smokeless tobacco, and e-cigarettes. The largest decreases in both exterior and interior advertising were in Category 1 cities, with more modest decreases, and some increases, in Category 2 cities. This suggests that while the flavored tobacco policies did not include restrictions on advertising, limits on flavored product availability were accompanied by decreases in tobacco advertising without compensatory increases in non-flavored tobacco advertisements.

This indicates that restrictions on flavored tobacco products, in addition to decreasing appeal of the products themselves to young people, may also impact youth tobacco initiation by decreasing tobacco advertising at retail outlets. This is consistent with prior retail studies that found: (1) that Chicago stores compliant with a flavored tobacco policy also had decreased advertising [19], and (2) there was a decrease in tobacco retailer advertising in Massachusetts cities that had passed flavored tobacco sales policies [34]. This is a potentially important impact of flavor policies, given that tobacco advertising is known to cause youth smoking and other tobacco product initiation [35,36]. Since limitations on tobacco advertising are more difficult to enact in the US compared to other countries [37], it is worth considering if policies limiting flavored tobacco sales in the US may be an effective strategy to decrease youth tobacco advertising exposure.

Lastly, we found that Category 2 retailers had significantly greater availability of cigalikes, mod or tank vapes, flavored e-cigarettes, and e-liquids compared to Category 1 retailers. This demonstrates that restrictions on flavored tobacco products reduce access to devices and liquids used for vaping, and further supports the idea that flavored products draw customers rather than vaping itself. While vaping products can easily be purchased on the Internet, at least one study has shown that young people were more likely to obtain e-cigarettes in physical stores (75% compared to 6% who purchased online) [38]. Reduced availability in retail stores may therefore discourage youth initiation and casual use that may lead to longer term nicotine addiction.

Our study ascertained retail product availability by utilizing independent trained auditors, which has the advantage of more objective measures of policy adherence than subjective reports or self-certification. The study also reflects random sampling of retailers across San Francisco and Alameda counties, independent of factors that may affect policy implementation. The total number of retailers in both counties declined by nearly 20% between our two waves of data collection, which prevented our ability to re-visit retailers from 2015 in a systematic way that would have allowed longitudinal assessment.

The impact of SF’s flavor policy on behavior has been the subject of two cross-sectional studies. One study of 247 young adults in San Francisco found decreases in overall and flavored tobacco use after the policy was implemented. This study also reported that among the younger (18–24-year-old) part of the study sample, flavored e-cigarette and flavored cigar use decreased significantly, but cigarette smoking increased [39]. This study relied on a small online convenience sample, participants in Amazon Mechanical Turk (MTurk), and participants’ retrospective recall of their product use approximately one year prior to the study. An analysis of YRBSS data from high schools between 2011–2019 comparing San Francisco schools to seven other districts found increased cigarette smoking in San Francisco [40]. However, this study had only a single data point following the flavored tobacco policy, and while the author attributed the increase in smoking to decreased flavored e-cigarette use, the paper did not report data on e-cigarette use. Research linking retail availability of flavored tobacco products and tobacco use behavior using a longitudinal design and population-based sampling would help to clarify the impact of the policy on behavior.

The findings of this study are subject to limitations. Our random sample of retailers from San Francisco and Alameda counties was designed to reflect the residential locations of our young adult population-based study, not the policy categories in this analysis. Thus, the analysis was limited to policy categories, and not all cities had sufficient representation in the sample to allow analysis by city. Nevertheless, as the young adults in our survey were themselves randomly selected and resided across all municipalities and regions in the two counties, and because the retailers were drawn first from a random sample in those areas, and second, from a random sample across the two counties, we expect that the probabilities of selection were very similar at both time points. What varied between 2015 and 2019–2020 was the number of tobacco retailers in business; there were 646 fewer tobacco retailers, a decrease of 38%, between the two data collection periods. While the auditors were well trained, they may not perfectly assess the retail availability of different products. Finally, as noted above, retail availability does not reflect tobacco use behavior.

## 5. Conclusions

In a robust sample of retailers in San Francisco and Alameda Counties before and after implementation of flavored tobacco policies, we found significant decreases in flavored product availability and in tobacco advertising in locations where policies were implemented. Comprehensive policies eliminating flavored tobacco sales are associated with significant decreases in flavored product availability and advertising, with greater effects in communities with more comprehensive implementation.

## Figures and Tables

**Table 1:** Prevalence of flavored tobacco product availability in San Francisco Bay Area retail stores over time and by flavored tobacco sales policy category, 2014–2020 San Francisco Bay Area Young Adult Health Surveys.

	Policy Category										
	(1) Flavored Tobacco Sales Policy Enacted between Wave 1 & Wave 2					(2) No Flavored Tobacco Sales Policy at Wave 2					
	2015		2019–2020			2015		2019–2020			
Product Availability	*n* = 206		*n* = 240			*n* = 36		*n* = 49			
Newport menthol cigarettes	89.9%	[0.86,0.94]	19.6%	[0.14,0.25]	***	85.3%	[0.73,0.97]	89.6%	[0.81,0.98]		+++
Little cigars	79.4%	[0.74,0.85]	79.2%	[0.74,0.84]		79.4%	[0.66,0.93]	83.7%	[0.73,0.94]		
Flavored little cigars (% of little cigars)	90.5%	[0.86,0.95]	22.4%	[0.16,0.28]	***	88.9%	[0.77,1.01]	95.1%	[0.89,1.02]		+++
Smokeless tobacco	42.7%	[0.36,0.50]	41.7%	[0.35,0.48]		55.9%	[0.39,0.73]	79.6%	[0.68,0.91]	*	+++
Flavored smokeless tobacco (% of smokeless)	83.5%	[0.76,0.91]	25.0%	[0.17,0.33]	***	89.5%	[0.76,1.03]	97.4%	[0.92,1.02]		+++
E-cigarettes	58.3%	[0.51,0.65]	55.8%	[0.50,0.62]		76.5%	[0.62,0.91]	77.6%	[0.66,0.89]		+++
Vape pens	19.2%	[0.14,0.25]	9.4%	[0.04,0.14]	*	23.5%	[0.09,0.38]	11.4%	[0.01,0.22]		
Blu menthol e-cigarette	53.3%	[0.44,0.62]	6.2%	[0.02,0.10]	***	64.3%	[0.47,0.82]	34.3%	[0.19,0.50]	*	++

*** *p* < 0.001, * *p* < 0.05. +++ Significantly more availability in policy category 2, *p* < 0.001; ++ *p* < 0.01.

**Table 2:** Prevalence of flavored tobacco advertising in San Francisco Bay Area retail stores over time and by flavored tobacco sales policy category, 2014–2020 San Francisco Bay Area Young Adult Health Surveys.

	Policy Category										
	(1) Flavored Tobacco Sales Policy Enacted between Wave 1 & Wave 2					(2) No Flavored Tobacco Sales Policy at Wave 2					
	2015		2019–2020			2015		2019–2020			
Exterior Advertising	*n* = 206		*n* = 236			*n* = 36		*n* = 49			
Non-menthol cigarette ads	47.6%	[0.41,0.54]	25.4%	[0.20,0.31]	***	52.8%	[0.36,0.69]	42.9%	[0.29,0.57]		+
Menthol cigarette ads	39.3%	[0.33,0.46]	6.4%	[0.03,0.09]	***	38.9%	[0.23,0.55]	26.5%	[0.14,0.39]		+
Little cigar ads	25.7%	[0.20,0.32]	12.3%	[0.08,0.16]	***	16.7%	[0.04,0.29]	20.4%	[0.09,0.32]		
Cigar ads	6.8%	[0.03,0.10]	3.0%	[0.01,0.05]		11.1%	[0.01,0.21]	6.1%	[−0.01,0.13]		
Smokeless tobacco ads	8.3%	[0.04,0.12]	5.1%	[0.02,0.08]		19.4%	[0.07,0.32]	12.2%	[0.03,0.21]		++
E-cigarette ads	32.0%	[0.26,0.38]	9.7%	[0.06,0.14]	***	41.7%	[0.26,0.58]	20.4%	[0.09,0.32]	*	
JUUL ads ^			14.0%	[0.10,0.18]				16.3%	[0.06,0.27]		
Other pod vape ads ^			3.8%	[0.01,0.06]				16.3%	[0.06,0.27]		+++
**Interior Advertising**											
Tobacco ads	60.9%	[0.54,0.68]	40.4%	[0.34,0.47]	***	50.0%	[0.34,0.66]	79.2%	[0.68,0.91]	**	++
E-cigarette ads	39.6%	[0.33,0.46]	19.3%	[0.14,0.24]	***	36.1%	[0.20,0.52]	41.7%	[0.28,0.56]		

*** *p* < 0.001, ** *p* < 0.01, * *p* < 0.05. +++ Significantly more ads in policy category 2, *p* < 0.001; ++ *p* < 0.01; + *p* < 0.05. ^ JUUL/other pod vapes were not available in tobacco retail stores in 2015.

**Table 3:** Prevalence of ENDS availability in Bay Area retail stores by flavored tobacco sales policy category, 2019–2020 San Francisco Bay Area Young Adult Health Surveys.

	Policy Category				
	(1) Flavored Tobacco Sales Policy Enacted between Wave 1 & Wave 2		(2) No Flavored Tobacco Sales Policy at Wave 2		
	2019–2020 (*n* = 161)				
Product Availability					
Cigalikes	46.8%	[0.38,0.56]	64.9%	[0.49,0.80]	*
Mods/tanks	10.6%	[0.05,0.16]	21.1%	[0.08,0.34]	*
Flavored e-cigarettes	21.3%	[0.14,0.29]	86.8%	[0.76,0.98]	***
Any JUUL products	91.4%	[0.87,0.96]	84.2%	[0.73,0.96]	
JUUL devices (% of Any)	73.7%	[0.66,0.81]	80.6%	[0.68,0.93]	
JUUL 4-packs (% of Any)	85.5%	[0.79,0.92]	85.3%	[0.73,0.97]	
Pod vapes (other than JUUL)	82.9%	[0.76,0.90]	89.5%	[0.80,0.99]	
E-liquids	12.7%	[0.07,0.18]	23.7%	[0.10,0.37]	*

*** *p* < 0.001, * *p* < 0.05.

## Data Availability

The data used in this study are embargoed until 2023. Details regarding the data can be obtained by emailing the corresponding author.

## Supplementary Materials

The following supporting information can be downloaded at https://www.mdpi.com/article/10.3390/ijerph19063455/s1, Table S1: Flavored Tobacco Sales Policies by City, Alameda and San Francisco Counties.
