# Supplementary material for: Flavored Tobacco Sales Restrictions Reduce Tobacco Product Availability and Retailer Advertising
Source: Int J Environ Res Public Health. 2022 Mar 15;19(6):3455. doi: 10.3390/ijerph19063455 (PMC8953832; doi:10.3390/ijerph19063455)
Supplement: Supplementary file 1 [file ijerph-19-03455-s001.zip › ijerph-1601106-supplementary.pdf]

Supplemental Table S1. Flavored Tobacco Sales Policies by City, Alameda and San Francisco Counties

City	2015 (N)	2019-20 (N)	Enacted/Modified	Policy Details
Hayward*	20	13	7/7/2014	Restricted flavored tobacco sales for new tobacco licenses only; stores already selling tobacco were exempted
San Francisco	137	103	7/21/2018	Terminated sales of all flavored tobacco products, including menthol
Alameda	3	8	7/1/2019	
Albany	1	0	10/15/2019	
Fremont	9	6	12/31/2019	
Livermore	8	8	1/1/2020	
San Leandro	15	13	8/5/2018	Terminated sales of all flavored tobacco products, **except** menthol
Berkeley	0	6	2017/2020	**2017**: No flavored tobacco sales within 600 feet of schools; **April 1, 2020**: Termination of all flavored tobacco sales
Oakland	66	59	2018/2020	**2018**: Termination of flavored tobacco sales, except in stores with >=60% tobacco sales; **May 12, 2020**: Eliminated exception
Dublin	2	4	3/14/2020	Terminated sales of all flavored tobacco products, including menthol
Castro Valley	7	3	9/6/2020	
San Lorenzo	3	1	9/6/2020	
Sunol	0	1	9/6/2020	
Pleasanton	3	3	1/1/2021	
Hayward*	20	13	1/14/2021	
Newark	9	11		No policy as of June 2021
Emeryville	1	0		
Union City	5	3		
